# Postoperative Management of Kirschner-Wire Fixation of All Phalangeal and Metacarpal Fractures at a Single Tertiary Care Center: A Retrospective Review

**DOI:** 10.1177/22925503251363056

**Published:** 2025-08-08

**Authors:** Andrew T. Chen, Carolyn Wang, Victor Ripan, Elena Huang, Avalon O’Connor, Patrick J. Kim, Emily Dunn, Achilleas Thoma, Helene Retrouvey

**Affiliations:** 1 Department of Surgery, Division of Plastic Surgery, 3710 McMaster University, Hamilton, ON, Canada; 2 Michael G DeGroote School of Medicine, 3710 McMaster University, Hamilton, ON, Canada; 3 Department of Health Research Methods, Evidence and Impact (HEI), 3710 McMaster University, Hamilton, ON, Canada

**Keywords:** hand fractures, rehabilitation, Kirschner-wires, pinning, brochage, broche de Kirschner, fractures de la main, réadaptation

## Abstract

**Introduction:** Kirschner-wire (K-wire) fixation (KF) is the most common operative technique for hand fractures among Canadian plastic surgeons. However, postoperative rehabilitation varies widely and rely on low-quality studies and expert opinion. The study reviewed KFs of phalangeal and metacarpal fractures at a single academic center to quantify practice variation and patient outcomes. **Methods:** This retrospective chart review analyzed all cases of KF of isolated phalangeal and metacarpal fractures performed by all plastic surgeons at a single tertiary care center in the last 7 years. The primary outcome was the duration from operation to K-wire removal. Secondary outcomes included the time from KF to range of motion (ROM) initiation, the interval between K-wire removal and ROM initiation, postoperative complications, and functional outcomes. **Results:**Among 289 patients, mean time from KF to K-wire removal was 28.6 days (SD 8.2). There was a high variability among the surgeons, with the shortest duration averaging 26 ± 6 days and the longest averaging 33.7 ± 8.6 days (*P* < .001). Mean time to initiation of ROM was similar across surgeons (mean 25.2 ± 11.4 days). Postoperative complications occurred in 26 patients (10.9%), primarily pin site infections (6.7%). Early versus late K-wire removal did not affect complication rates. There were no differences in functional outcomes between surgeons. **Conclusion:** There is considerable variability in postoperative rehabilitation following KF of hand fractures among surgeons at a single academic center. Our study suggests that supervised ROM can be initiated safely as early as 3 weeks postoperatively, regardless of whether K-wires remain in situ.

## Résumé

**Introduction **: La fixation par broche de Kirschner (broche K; FK) est la principale technique opératoire pour les fractures de la main chez les plasticiens canadiens. Cependant, la réadaptation postopératoire varie considérablement et repose sur des études de faible qualité et des avis d’experts. L’étude a permis de passer en revue les FK des fractures phalangiennes et métacarpiennes dans un seul centre universitaire pour quantifier les variations selon les pratiques et les résultats cliniques des patients. **Méthodologie :** Cette analyse rétrospective des dossiers a permis d’analyser tous les cas de FK des fractures phalangiennes et métacarpiennes isolées effectuées par tous les plasticiens d’un seul centre de soins tertiaires au cours des sept années précédentes. Le résultat primaire était la période entre l’opération et le retrait de la broche K. Les résultats secondaires incluaient la période entre la FK et le début de l’amplitude de mouvements (AdM), les complications postopératoires et les résultats fonctionnels. **Résultats :** Chez les 289 patients, la période moyenne entre la FK et le retrait de la broche K était de 28,6 jours (ÉT 8,2). La variabilité entre les chirurgiens était très élevée, la période la plus courte étant d’une moyenne de 26 ± 6 jours et la plus longue, d’une moyenne de 33,7 ± 8,6 jours (*P* < 0001). La période moyenne avant la reprise de l’AdM était semblable entre les chirurgiens (moyenne de 25,2 ± 11,4 jours). Des complications postopératoires ont été observées chez 26 patients (10,9%), principalement en raison d’infections au foyer de la broche (6,7%). Le retrait précoce plutôt que tardif de la broche K n’avait pas d’effet sur les taux de complication. Les résultats fonctionnels ne variaient pas selon les chirurgiens. **Conclusion :** La réadaptation postopératoire est très variable après une FK des fractures de la main chez les chirurgiens d’un seul centre universitaire. L’étude indique que l’AdM supervisée peut être entreprise en toute sécurité dès trois semaines après l’opération, que les broches K demeurent en place ou non.

## Introduction

Hand fractures are very common and account for up to 20% of all fractures of the body.^1^ Of these, metacarpal fractures account for up to 50% and phalanges account for up to 42%.^2^ For most fracture patterns, conservative management, such as with splinting±closed reduction yields excellent functional results.^3,4^ For unstable fractures, nonoperative management may result in malunion or nonunion causing clinical shortening, malrotation, or incomplete extension.^5^ To address and/or prevent these, surgical intervention is often required.

Hand surgeons can utilize a variety of methods to stabilize fractures including Kirschner wires (K-wires), lag screws, intramedullary screws, or rigid plates.^6^ Closed reduction (CR) and percutaneous pinning using K-wires is the most minimally invasive surgical option for treatment of phalangeal and metacarpal fractures.^7,8^ It is the most common method for surgical fixation among Canadian plastic surgeons.^9^ In certain fracture patterns that are difficult to achieve CR, open reduction (OR) may be used to visualize the fracture and achieve reduction prior to K-wire fixation (KF).

Following surgical fixation, appropriate timing and patient motivation for postoperative rehabilitation are some of the main contributors to improved hand function.^10^ With conservative management, cast/splint immobilization is used to prevent malunion or nonunion during callous formation in the first 3 to 6 weeks after injury.^11^ With the addition of K-wires, increased biomechanical stability may result in improved alignment and in some cases allow for earlier protected ROM.^12^ However, even with stable fixation, the use of early postoperative rehabilitation protocols varies, with some surgeons initiating ROM as early as 1 to 2 weeks and others delaying until 4 to 6 weeks.^13^ Principles of fracture immobilization after surgical fixation are based on historical studies with few high-quality studies to provide guidance.^14–16^ Thus, there is little evidence to guide duration of immobilization which results in significant variation in surgical practice.

We hypothesized that there is significant practice variation with total immobilization time after KF between surgeons. We aimed to review all CR/ORKF of phalangeal and metacarpal fractures of plastic surgeons at a single academic center over the past 7 years to quantify the variation in practice and patient outcomes.

## Methods

This was a retrospective chart review of all CR/ORKF of isolated phalangeal and metacarpal fractures by all plastic surgeons at a single tertiary care center from January 2018 to April 2024. This study was approved by the institution's ethics board prior to commencement (ID Reference: 2024-17567-C). This study followed STROBE guidelines.^17^

### Patient Selection

The cases were identified using Ontario Health Insurance Plan (OHIP) billing codes for KF of phalangeal or metacarpal fractures (Table S1). Medical records of identified patients were accessed to confirm eligibility. All data were extracted from our local electronic medical records. Inclusion criteria included all patients ≥18 years of age with single- or double-digit, closed, isolated, metacarpal, proximal phalanx, middle phalanx, or distal phalanx fractures that underwent CR/ORKF. There must be a minimum of 1 postoperative visit. We excluded pediatric patients (<18 years of age), open fractures, associated nerve, vessel, tendon, or ligament injuries, concomitant upper extremity injuries, and any records with insufficient information to assess the primary outcome. We excluded patients seen in our institution's designated Workplace Safety and Insurance Board (WSIB) clinic.

### Outcome Measures

We collected data including patient demographics (age, hand dominance, history of smoking and diabetes, occupation), injury date, and date of plastic surgery consultation. We extracted injury characteristics including digit, bone injured (proximal phalanx, middle phalanx, distal phalanx, metacarpal), injury location (shaft, base, neck), fracture pattern (transverse, oblique, comminuted), and joint involvement. We extracted surgeon names, which were then de-identified and coded as Surgeon 1, 2, …, 6. Operative details were extracted (date of surgery, number of wires, operative time, anesthetic used, location including minor procedure room or operative room). Operative outcomes were extracted including follow up dates, occupational therapy visits, type of splint (thermoplastic vs conventional), immobilization time, date of K-wire removal, date beginning ROM, complications (infection, malunion, nonunion, and others), functional outcomes (pain, return to work, functional status, and total AROM), and reoperation rate. Data was extracted from plastic surgery and hand therapy notes. Pain was categorized as none, mild, moderate, and severe according to hand therapy notes. Return to work was defined as the first plastic surgery or hand therapy note that reported that the patient was back to work. Functional status was categorized as independent, independent with some assistance, or requiring assistance for most activities. AROM was measured using hand goniometer at the PIP, DIP, and MCP joints and total AROM was calculated as the sum of the angles at maximum active flexion. Data was extracted into a Microsoft Excel spreadsheet created a priori (Version 16.16.13 Microsoft, Redmond, WA, USA).

Our primary outcome was average duration (in days) from KF to K-wire removal. Secondary outcomes included average duration (in days) from KF to beginning ROM exercises, duration of KF based on patient and injury characteristics, average duration (in days) from K-wire removal to beginning ROM exercises, postoperative complication rates, and functional outcomes. Time to beginning ROM exercises was determined by the first clinic note or hand therapy note that reported that the patient was able to begin ROM exercises.

### Data Analysis

The demographics of included patients, surgeon practice, and postoperative complications were reported using descriptive statistics. Continuous variables were summarized using mean and standard deviation (SD) or mean and range, where applicable. Categorical variables were summarized using number of patient (*n*) and percentages (%). Two-sided Student's T-test and analysis of variance (ANOVA) were used to determine if there is a significant difference in average time from KF to removal of hardware, and from KF to beginning ROM. This was presented as mean differences (MD), which refers to the difference between the averages of two surgeons. We compared total immobilization duration based on the fractured bone and other injury characteristics using ANOVA. We compared the proportion of patient characteristics and injury patterns between surgeons using the Chi-square and Fisher's two-sided test. A *P* value < .05 was considered significant. Statistical analysis was conducted on R Studio (Version 4.4.2) and GraphPad Prism (Version 8.0.0).

## Results

### Patient Demographics

There were 356 patient charts identified, of which 117 were excluded based on our exclusion criteria, most commonly because of associated tendon, ligament, or vessel injury and open fractures (Table S2). Less than 5% of patients were excluded due to insufficient information. We included 239 patients, of whom 176 (73.6%) underwent CRKF and 63 (26.4%) underwent ORKF. Demographic details are presented in Table 1. The mean age was 41.8 years (SD 16.6), 68.9% were male, and 92.5% were right-hand dominant. Sixty-one (28.8%) patients were current smokers, and 20 (9.4%) were previous smokers. Twenty (9.4%) were diabetic. The average time from injury to plastic surgery consult was 3.9 days (SD 6.3) and from plastic surgery consult to operation was 2.7 days (SD 3.2). Overall, the average time from injury to surgical operation was 6.6 days (SD 7.1).

**Table 1.** Patient Characteristics.

Age (at OR)		Mean	SD
	Age	41.8	16.6
Sex		*N*	%
	Male	146	68.9
	Female	93	43.9
Handedness		*N*	%
	Right	196	92.5
	Left	21	9.9
	Not reported	22	10.4
Diabetes		*N*	%
	Yes	20	9.4
	No	219	103.3
Smoking		*N*	%
	Never	142	67
	Current smoker	61	28.8
	Ex-smoker	20	9.4
	Not reported	16	7.5
Time (*d*) between injury and hospital visits		Mean	SD
	Days from injury to plastic surgery consult	3.9	6.3
	Days from plastic surgery consult to OR	2.7	3.2
	Days from injury to OR	6.6	7.1

### Impact of Injury Characteristics on Duration of K-Wire Fixation

**Figure 1.**
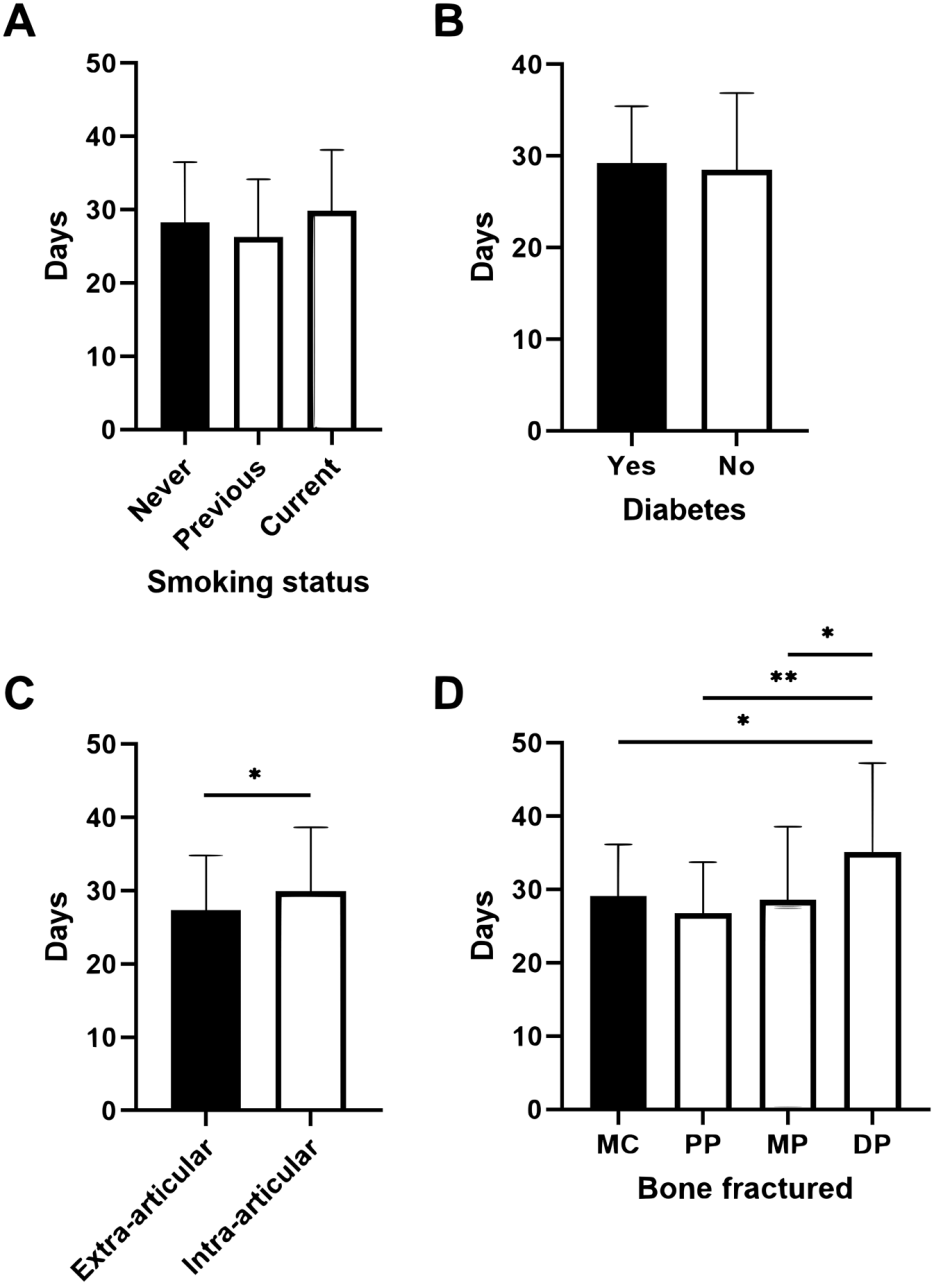
Variation in duration of K-wire fixation based on: (A) patient smoking status, (B) presence of diabetes, (C) intra-articular involvement, and (D) bone involved. MC = metacarpal, PP = proximal phalanx, MP = middle phalanx, DP = distal phalanx.

Injury characteristics are summarized in Tables 2 and 3. The most commonly injured bone was the metacarpals (46.7%), followed by the proximal phalanges (30.8%). The most common digit injured was the little finger (41.7%). There was no significant difference in the length of KF based on smoking status (Figure 1A) and diabetes (Figure 1B). There was no significant difference in the length of KF based on comminution (28.6 vs 28.7 days). There was a significant difference in immobilization time with KF between distal phalanx and middle phalanx (MD 6.5 ± 6.2, *P* = .04), proximal phalanx (MD 8.4 ± 5.9, *P* = .002), and metacarpals (MD 6.0 ± 5.8, *P* = .04) (Figure 1D). There was a significant difference between the length of KF of intra-articular and extra-articular fractures (30.0 vs 27.4, MD 2.6 ± 1.1, *P* = .02) (Figure 1C). There was no significant difference between the proportion of patients with intra-articular fractures (*P* = .13) or the type of bone involved (*P* = .99) between surgeons. Thirty-seven (15.5%) patients had a second fracture. The length of fixation was not significantly different between those who had 1 or 2 fractures (*P* = .37).

**Table 2.** First Fracture Characteristics (*N* = 239).

Hand		*N*	%
	Right	136	56.9
	Left	103	43.1
Bone		*N*	%
	Distal phalanx	15	6.3
	Middle phalanx	43	18
	Proximal phalanx	78	32.6
	Metacarpal	103	43.1
Digit		*N*	%
	D1	33	13.8
	D2	22	9.2
	D3	25	10.5
	D4	61	25.5
	D5	98	41
Intraarticular/Extraarticular		*N*	%
	Extra	124	51.9
	Intra	115	48.1

**Table 3.** Injury Characteristics of Patients With Second Fractures (*N* = 37).

Total		37	
Hand		*N*	%
	Right	27	73
	Left	10	27
Bone		*N*	%
	Distal phalanx	1	0.4
	Middle phalanx	3	1.3
	Proximal phalanx	7	2.9
	Metacarpal	26	10.9
Digit		*N*	%
	D1	0	0
	D2	2	0.8
	D3	3	1.3
	D4	15	6.3
	D5	17	7.1
Intraarticular/Extraarticular		*N*	%
	Extra	27	73
	Intra	10	27

### Variation in Immobilization Duration Among Surgeons for all Fracture Types

**Figure 2.**
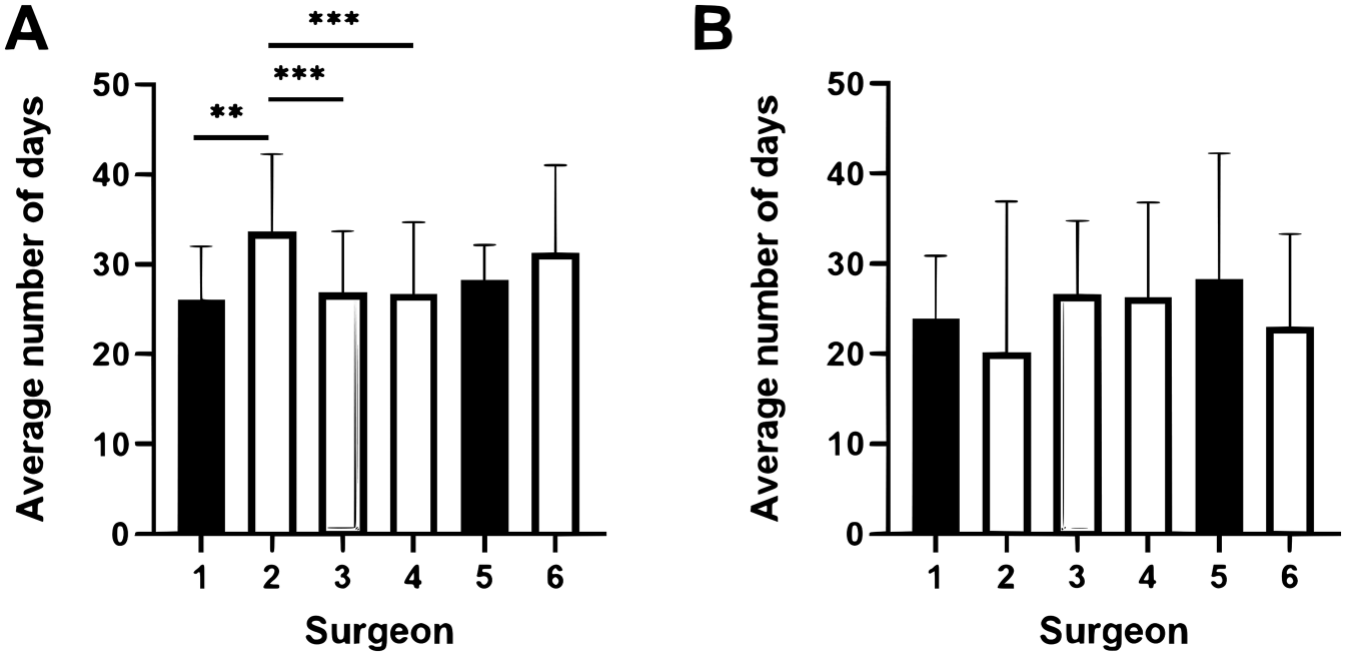
Variation in duration of immobilization between surgeons at a single tertiary care hospital. (A) Average length of time in days from operation to Kirschner wire removal. (B) Average length of time in days from operation to beginning range of motion.

The average time from KF to K-wire removal was 28.6 days (SD 8.2), and the average time from KF to beginning ROM was 25.2 days (SD 11.4). There was a significant variation in the duration of KF between surgeons. Surgeon 1 had the shortest duration of KF averaging 26 ± 6 days and Surgeon 2 had the longest duration averaging 33.7 ± 8.6 days (MD 7.7 ± 6.2, *P* < .001) (Figure 2A). There was no difference in the time to beginning ROM between surgeons (Figure 2B). Most patients began ROM immediately or shortly after pin removal. Fifty-five (23%) patients began ROM prior to pin removal. Surgeon 2 had a larger proportion of patients begin ROM prior to pin removal (63.4%) compared to other surgeons (14.6%). Eighty-seven (36.4%) patients saw our hospital-affiliated hand therapy. Most patients (63.2%) received a thermoplastic splint in their first hand therapy appointment (Table S3).

We reviewed all patients who had prolonged KF (>6 weeks) and early pin removal (<3 weeks) to further assess possible reasons for variation in immobilization duration. Sixteen patients had prolonged KF (>6 weeks). Of these, 3 missed their pin removal appointment, 1 had an X-ray that demonstrated delayed healing, and the remainder did not provide comments. Twenty-three patients had K-wire removal within 3 weeks. The majority was due to the surgeon preference to reduce stiffness with a plan for removal between 2 and 3 weeks. Six patients had them removed due to a pin site infection.

### Comparison of K-Wire Fixation and Immobilization Durations Among Surgeons for Metacarpal and Proximal Phalanx Fractures

**Figure 3.**
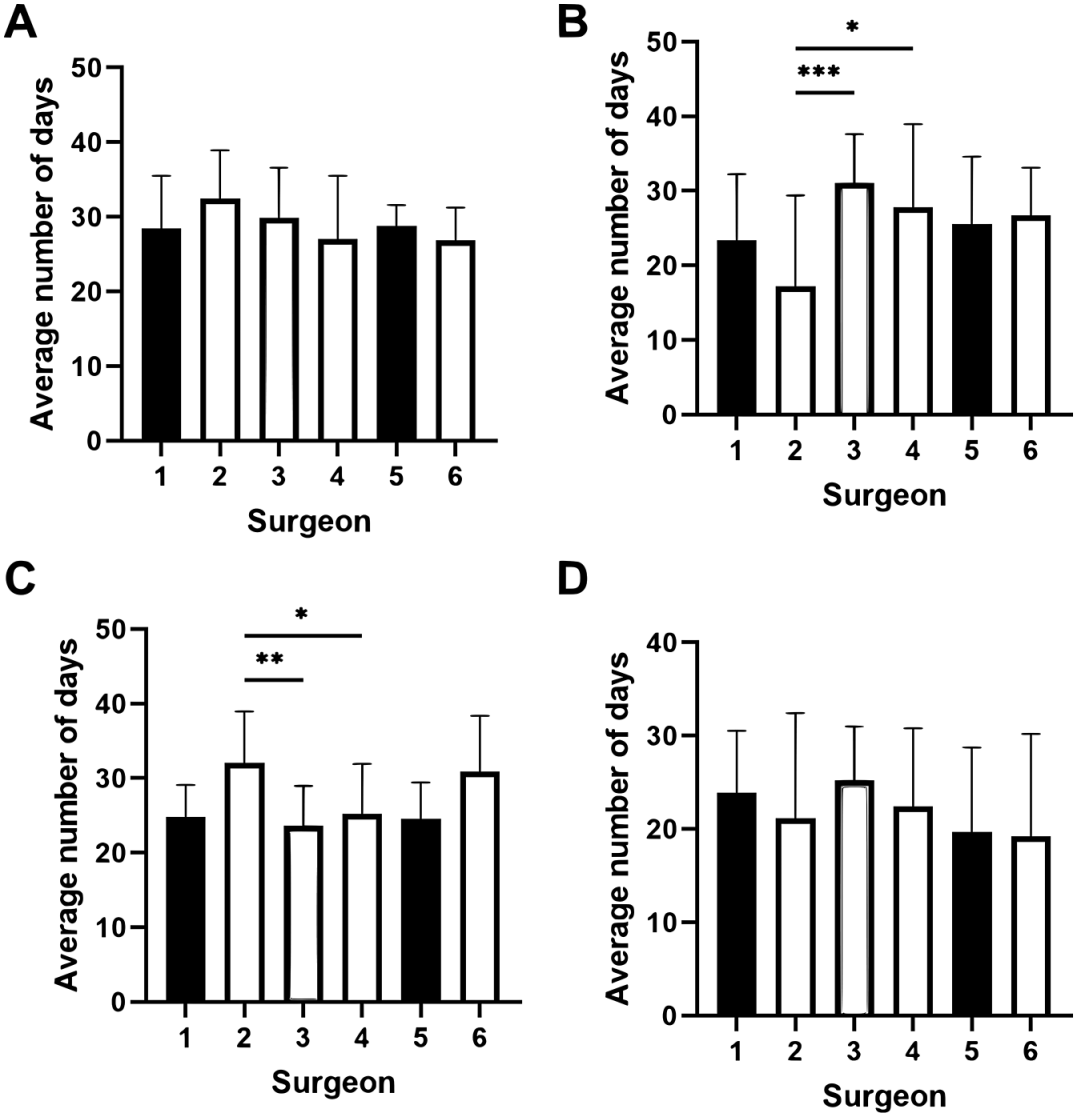
Variation in duration of K-wire fixation and immobilization between surgeons in metacarpal and proximal phalanx fractures. (A) Among all metacarpal fractures, average duration from K-wire fixation to removal and (B) beginning ROM. (C) Among all proximal phalanx fractures, average duration from K-wire fixation to removal and (D) beginning ROM.

We performed subgroup analysis for the 2 most common bone fractures, metacarpal and proximal phalanx fractures. For metacarpal fractures, there was no significant difference in duration of KF fixation between surgeons (Figure 3A). There was a significant difference in the time from KF fixation to beginning ROM with Surgeon 2 (17.2 ± 12.2) having the shortest duration and Surgeon 3 (31.0 ± 6.4) having the longest duration (MD −13.8 ± 9.22, *P* < .0001) (Figure 3B). For proximal phalanx fractures, Surgeon 2 (32.0 ± 7.0) had the longest duration of KF and Surgeon 3 (23.6 ± 5.2) had the shortest (MD 8.4 ± 6.8, *P* < .01), but there was no significant difference in duration from KF to beginning ROM (22.6 ± 8.4)

### Postoperative Complications

There was a total of 26 (10.9%) postoperative complications, most commonly being pin site infection (16, 6.7%) (Table 4). All postoperative infections required antibiotics, and none required admission. Six patients required early K-wire removal due to infection. Two patients required reoperation for malunion due to the development of secondary chronic deformities. One patient had delayed healing following a pilon fracture that required long-term follow-up and hand therapy. There was no difference in complication rate with earlier (<4 weeks) versus later (≥4 weeks) pin removal (10.9% vs 11.7%).

**Table 4.** OR Characteristics and Complications.

Wires used	*N*	%
1	10	4.2
2	169	70.7
3	30	12.6
4	26	10.9
5	3	1.3
6	1	0.4
Wire placement	*N*	%
Buried	22	9.2
Outside	217	90.8
Procedure	*N*	%
CRKF	176	73.6
ORKF	63	26.4
Complication	*N*	%
No	213	89.1
Yes	26	10.9
Complication	*N*	%
Infection	16	6.7
Malunion	7	2.9
Nonunion	0	0
Other	3	1.3
Reoperation	*N*	%
No	237	99.2
Yes	2	0.8
Infection requiring antibiotics	*N*	%
No	0	0
Yes	16	100

### Functional Outcomes

**Figure 4.**
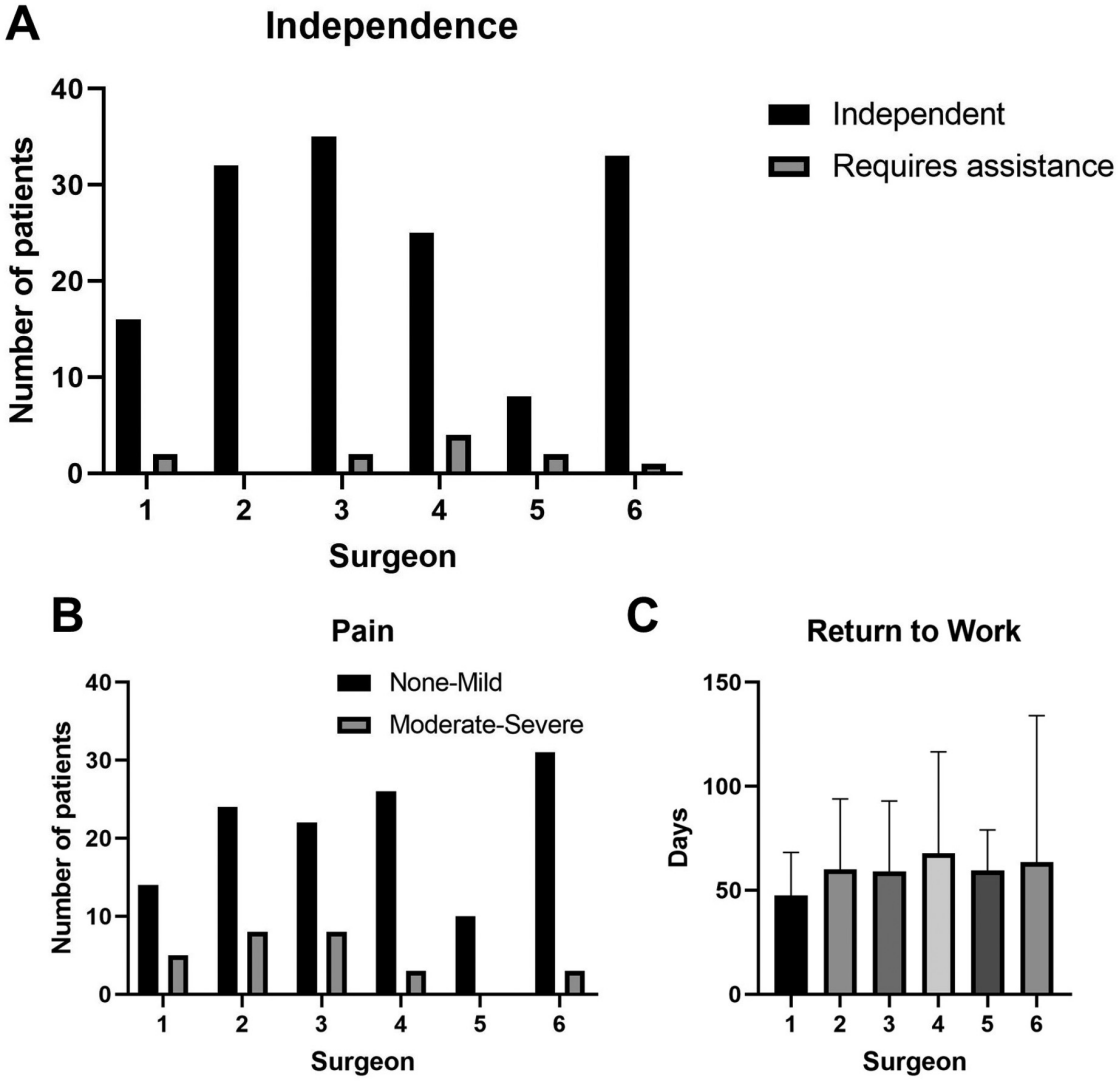
Functional outcomes between surgeons. (A) Patient independence with activities of daily living, (B) pain, and (C) time to return to work.

Overall, 149 patients (93.1%) of patients reported independent function in/out of splint, 11 (6.9%) patients reported some difficulty, and no patients reported needing assistance for most activities (Figure 4A). There was no significant difference in functional status between surgeons. There were 68 (44.4%) patients who reported no pain, 59 (38.6%) reported mild pain, 17 (11.1%) reported moderate pain, and 9 (5.9%) reported severe pain. There was no significant difference in pain between surgeons (Figure 4B). The median time to return to work was 60.4 days (range 3-223). There was no significant difference in time to return to work between surgeons (Figure 4C). There was no difference in initial or final total active ROM between surgeons.

## Discussion

In the present study, we present all CR/ORKF of isolated single- or double-digit hand fractures performed by 6 plastic surgeons at a tertiary care center. Here, we show a significant variation with time from KF to K-wire removal between surgeons, independent of patient or injury characteristics. There was no overall difference in the duration from KF to beginning ROM between surgeons. There was a low complication rate across surgeons, and the variation in KF duration did not appear to influence functional outcomes in this study.

This study suggests that there is a significant variation in the length of KF between plastic surgeons at a single site. Interestingly, although Surgeon 2 on average preferred a longer K-wire duration, they did not delay initiating ROM exercises, suggesting that they were comfortable with beginning ROM with K-wire in situ. Previous observational studies have reported a wide variation of protocols with K-wire fixation.^18–21^ Some surgeons prefer to immobilize for a longer period as aggressive ROM can lead to fracture displacement.^18^ In addition, delaying ROM until K-wires are removed has been suggested to avoid complications such as pin site infection, malunion, or non-union, which may be associated with K-wire exposure and manipulation.^19^ However, other reports suggest that ROM can begin as early as 3 to 5 days once pain is adequately controlled.^20^ For instance, Del Chiaro et al began ROM out of splint for all proximal phalanges fractures 1 week after undergoing K-wire fixation.^21^ Overall, there is a lack of standardization, which is evidenced by the significant variation in postoperative surgical fixation and ROM protocols between surgeons even at a single tertiary care site.

We found that even within 1 plastic surgeon's practice, there was a high standard deviation in immobilization time, suggesting a high complexity of hand fracture patterns that require individualized postoperative rehabilitation plans.^22^ Injury characteristics, such as intra-articular extension and the type of bone involved, were significant factors for increased duration of KF. It is well understood that with phalangeal fractures, particularly proximal or middle phalanges that involve the proximal interphalangeal joint, early ROM has been recommended to avoid long-term stiffness.^16^ In other fracture patterns that are inherently unstable, a longer immobilization period may be needed to prevent malunion.^23^ Characteristics such as segmental bone loss, percentage of bone contact, method of K-wire fixation, and fractures with deforming forces may influence a surgeon's decision on when to remove K-wires.^18,24^ Therefore, while postoperative rehabilitation could be standardized to optimize patient outcomes, patient demographics, patient compliance, and injury characteristics need to be carefully considered.

We assessed the complication rate of K-wire fixation among all patients in our cohort. There was no difference in complication rate between those who had K-wires removed early or late, suggesting that K-wires left in situ for 3 to 6 weeks appear to be safe. We reported a 10.9% rate of complications, which was comparable to previous reports.^25,26^ In addition, we assessed functional outcomes to see if variation in K-wire fixation may influence patient outcomes. Although there was no significant difference between surgeons, significant reporting heterogeneity and missing data limited definitive conclusions. In previous studies, early protected ROM in patients with fifth metacarpal neck fractures has been shown to have improved functional outcomes compared to immobilization.^27^ However, whether similar findings could be found in select patients who begin earlier ROM after K-wire fixation is unclear. Standardized measures and patient-reported outcomes will be important to further identify patients who may benefit from early ROM after K-wire fixation. Overall, despite differences in duration of K-wire fixation, these protocols were safe and appeared to have similar complication profiles and functional outcomes.

The primary strength of this study is the long-term analysis of multiple surgeons’ practices at a single tertiary site. This allows for informative comparative analysis of how postoperative management of KF may vary between operating surgeons. However, there are some limitations to this study. Firstly, there was significant reporting heterogeneity and missing data with functional outcome measures. However, we extracted all hand therapy and plastic surgeon notes related to functional outcome. While definitive conclusions cannot be drawn from these results, it is beneficial for hypothesis generation and future studies. Further prospective studies using validated patient-reported outcomes, such as the Disabilities of Arm, Shoulder, and Hand questionnaire (DASH) or visual analogue scale (VAS) for pain, would provide further insight. Second, this study is limited by a small sample size. However, this was the largest available cohort size at our institution as electronic medical records were introduced in 2023, and records were only reliably available from 2018. In addition, this sample size was sufficiently powered and had a similar number of patients compared to previous studies.^28–30^ Lastly, this study is prone to limitations inherent to the study design of a retrospective review, such as inconsistent reporting, missing data, and observer bias. For instance, other important injury characteristics such as osteoporosis, steroid use, or nutrition status were not available but may influence K-wire fixation duration. However, we reviewed all available hand therapy and surgeon notes to extract information pertaining to our primary and secondary objectives. Further, we had a low exclusion rate based on missing data or inconsistent reporting.

## Conclusion

In the present study, we demonstrate that there is a high degree of variability with postoperative rehabilitation after KF of hand fractures between surgeons at a single tertiary care center, likely due to previous training experiences and practice patterns. There was a low rate of postoperative complications following CR/ORKF. Altogether, the above findings suggest that ROM can begin safely as early as 3 weeks. This does not seem to be limited by K-wire removal, and if there are concerns of delayed healing, ROM can begin with or without K-wires in situ. Given that several factors impact ROM, including the digit/bone involved, age, fracture characteristics, co-morbidities, and participation in postoperative rehabilitation, our next steps will be aimed at designing a prospective study to analyze functional outcomes based on the timing of KF.

## Supplemental Material

sj-docx-1-psg-10.1177_22925503251363056 - Supplemental material for Postoperative Management of Kirschner-Wire Fixation of All Phalangeal and Metacarpal Fractures at a Single Tertiary Care Center: A Retrospective Review Supplemental material, sj-docx-1-psg-10.1177_22925503251363056 for Postoperative Management of Kirschner-Wire Fixation of All Phalangeal and Metacarpal Fractures at a Single Tertiary Care Center: A Retrospective Review by Andrew T. Chen, Carolyn Wang, Victor Ripan, Elena Huang, Avalon O’Connor, Patrick J. Kim, Emily Dunn, Achilleas Thoma and Helene Retrouvey in Plastic Surgery

## Abbreviations

ANOVA: analysis of variance
CR: closed reduction
HiREB: Hamilton integrated research ethics board
KF: Kirschner wire fixation
K-wires: Kirschner wires
MD: mean difference
OR: open reduction
PP: percutaneous pinning
ROM: range of motion
SD: standard deviation
STROBE: The Strengthening the Reporting of Observational Studies in Epidemiology
