# Supplementary material for: Postoperative Management of Kirschner-Wire Fixation of All Phalangeal and Metacarpal Fractures at a Single Tertiary Care Center: A Retrospective Review
Source: Plast Surg (Oakv). 2025 Aug 8:22925503251363056. Online ahead of print. doi: 10.1177/22925503251363056 (PMC12334410; doi:10.1177/22925503251363056)
Supplement: sj-docx-1-psg-10.1177_22925503251363056 - Supplemental material for Postoperative Management of Kirschner-Wire Fixation of All Phalangeal and Metacarpal Fractures at a Single Tertiary Care Center: A Retrospective Review [file sj-docx-1-psg-10.1177_22925503251363056.docx]

**Supplemental Data**:

| OHIP Billing Code |  |
| --- | --- |
| F005 | Fractures - Phalanx - closed reduction |
| F006 | Fractures - Intra-articular - closed reduction |
| F007 | Fractures - Phalanx - open reduction |
| F009 | Fractures - Metacarpal - closed reduction |
| F010 | Fractures - Intra-articular - open reduction |
| F011 | Fractures - Metacarpal - open reduction |
| E569 | Percutaneous pinning, to closed reduction fee |
| E826 | Percutaneous pinning |

**Table S1: OHIP billing codes for chart identification**

| **Excluded** |  |
| --- | --- |
| >2 digits | 14 |
| Associated nerve, vessel, tendon, or ligament injury | 21 |
| Concomitant upper extremity injury | 14 |
| Insufficient information to assess primary outcome | 7 |
| Open fracture | 38 |
| No OR/Fracture | 6 |
| Duplicate | 2 |
| ORIF | 15 |
| **Total** | 117 |

**Table S2: Excluded patients**

| **Surgeon** | **1** | **2** | **3** | **4** | **5** | **6** | **Total** |
| --- | --- | --- | --- | --- | --- | --- | --- |
| **Average time from OR to 1st hand therapy (STD)** | 24.7 (13) | 11.2 (9.3) | 33.1 (57.2) | 20.7 (14.7) | 11.3 (8.1) | 12.7 (8.7) | 20.4 (32) |
| **Number of patients that did not see hospital hand therapy (%)** | 2 (10) | 5 (12.2) | 27 (39.7) | 40 (62.5) | 4 (36.4) | 9 (25.7) | 87 (36.4) |
| **Number of patients that began ROM prior to pin removal (%)** | 7 (35) | 26 (63.4) | 4 (5.9) | 6 (9.4) | 1 (9.1) | 11 (31.4) | 55 (23.0) |
| **Number of patients that received thermoplastic splint (%)** | 18 (90) | 34 (82.9) | 41 (60.3) | 23 (35.9) | 8 (72.9) | 27 (77.1) | 151 (63.2) |

**Table S3: Hand therapy characteristics**
